# Laparoscopic resection of schwannoma in the hepatoduodenal ligament: a case report

**DOI:** 10.1186/s40792-021-01271-y

**Published:** 2021-08-19

**Authors:** Tomoaki Bekki, Koichi Oishi, Takeshi Tadokoro, Yosuke Namba, Sho Okimoto, Shoichiro Mukai, Yasufumi Saito, Seiji Fujisaki, Toshihiro Nishida, Hideki Ohdan, Toshikatsu Fukuda

**Affiliations:** 1 Department of Surgery, Chugoku Rosai Hospital, Hirotagaya 1-5-1, Kure, Hiroshima, Japan; 2 Department of Pathology, Chugoku Rosai Hospital, Hiroshima, Japan; 3 Department of Gastroenterological and Transplant Surgery, Applied Life Sciences, Institute of Biomedical and Health Sciences, Hiroshima University, Kasumi 1-2-3 Minami-ku, Hiroshima, Japan

**Keywords:** Schwannoma, Hepatoduodenal ligament, Laparoscopic surgery

## Abstract

**Background:**

The occurrence of schwannomas in the hepatoduodenal ligament is rare, and its preoperative accurate diagnosis is difficult. Only few cases have been treated with laparoscopic surgery.

**Case presentation:**

A 54-year-old man visited our hospital following abnormal abdominal computed tomography findings. He had no complaints, and his laboratory investigations were normal. Abdominal contrast-enhanced computed tomography revealed a tumor with enhancement at the margin of the hepatoduodenal ligament. The abdominal magnetic resonance imaging findings of the tumor showed hypointensity on the T1-weighted images and mixed hypointensity and hyperintensity on the T2-weighted fat-suppression images. Positron emission tomography showed localized accumulation of fludeoxyglucose only in the hepatoduodenal ligament tumor. The patient underwent laparoscopic tumor resection for accurate diagnosis. Histopathologically, the tumor was mainly composed of spindle cells, which were strongly positive for S-100 protein on immunohistochemical staining. The patient was discharged without any postoperative complications on day 5.

**Conclusions:**

Complete tumor resection is essential for schwannomas to avoid recurrence. Laparoscopic surgery is useful for schwannomas occurring in the hepatoduodenal ligament and can be performed safely by devising an appropriate surgical method.

## Background

Schwannomas are mesenchymal neoplasms originating from the Schwann cells, which are the neuroglial cells surrounding the peripheral nerves [1]. They can occur in any part of the body, such as the head, neck, trunk, or extremities [1]. Retroperitoneal and gastric schwannomas are the most common types of schwannomas occurring in the abdominal cavity [2, 3]. However, schwannomas in the hepatoduodenal ligament are extremely rare, and only a few case reports have described their laparoscopic resection.

Herein, we present a case of laparoscopic resection of schwannoma in the hepatoduodenal ligament that was unexpectedly detected on abdominal computed tomography (CT).

## Case presentation

**Fig. 1:**
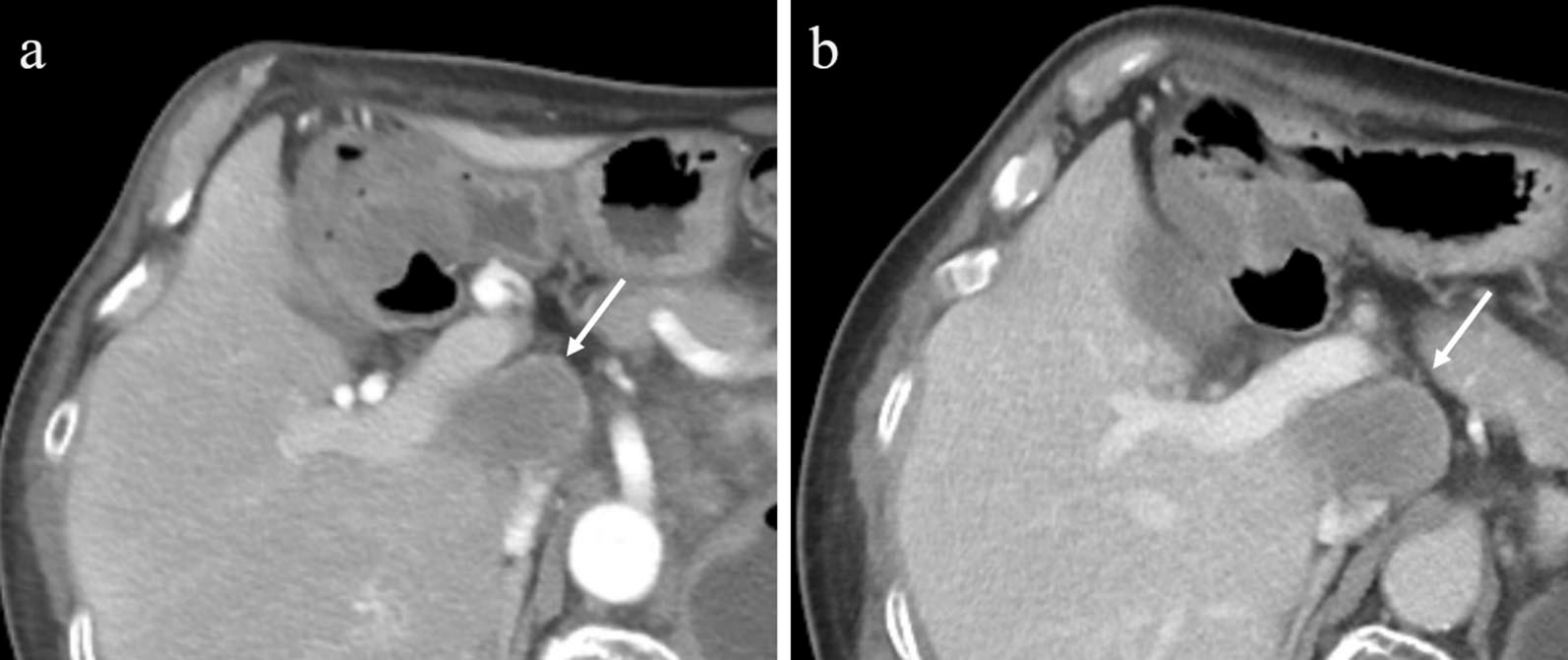
Abdominal contrast-enhanced computed tomography findings. **a** The tumor (white arrow) measuring 40 mm showed enhancement at the peripheral margin in the arterial phase. **b** The tumor (white arrow) with peripheral margin enhancement was prolonged in the delayed phase

**Fig. 2:**
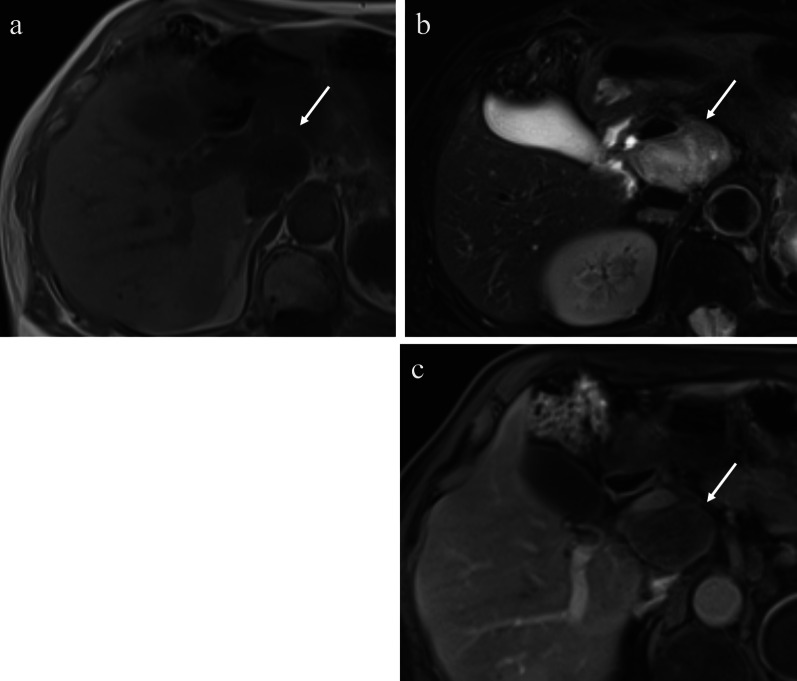
Abdominal magnetic resonance imaging findings. **a** The tumor (white arrow) showed hypointensity on T1-weighted images. **b** The tumor (white arrow) showed mixed hypointensity and hyperintensity on T2-weighted fat-suppression images. **c** Abdominal contrast-enhanced MRI revealed that the peripheral margin of the tumor (white arrow) was enhanced in the arterial phase. *MRI* magnetic resonance imaging

**Fig. 3:**
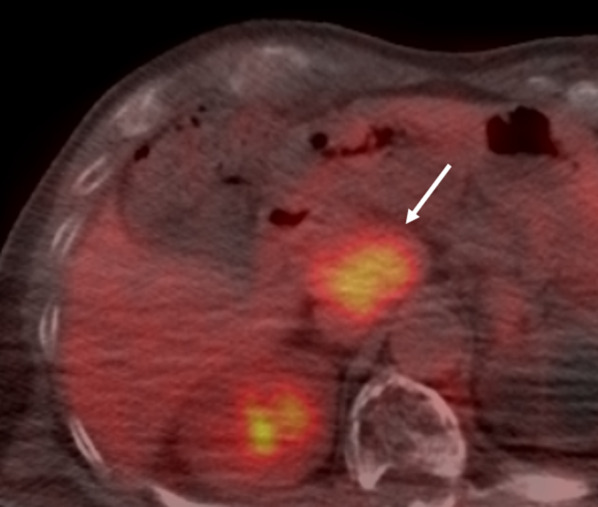
Positron emission tomography findings. The PET revealed localized accumulation of fludeoxyglucose (5.9 F) in the hepatoduodenal ligament tumor (white arrow). *PET* positron emission tomography

**Fig. 4:**
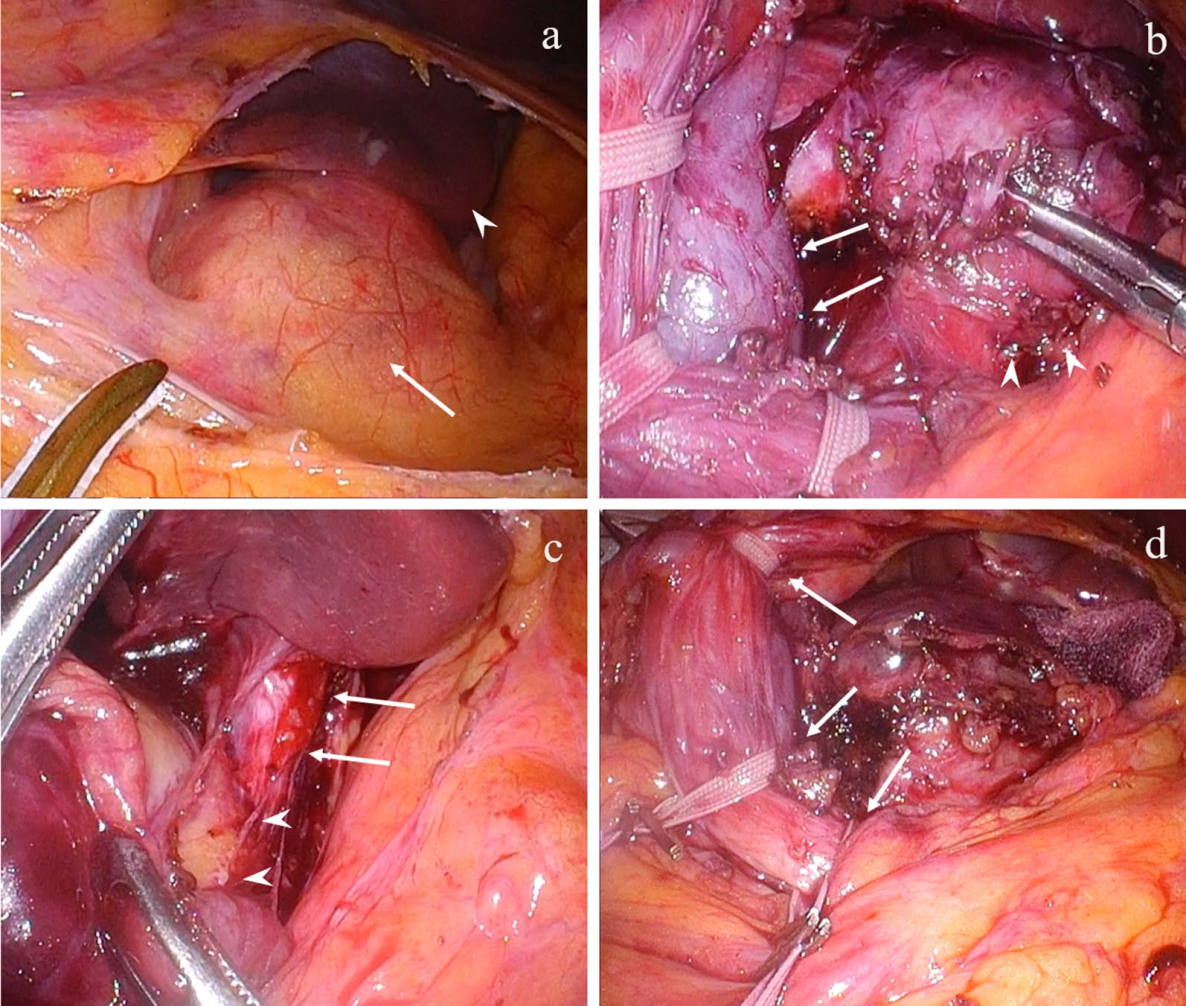
Intraoperative findings. **a** The first observation of the tumor (white arrow), before the resection, is observed. The tumor was located under the caudate lobe of the liver (white arrowhead).** b**, **c** The tumor (white arrowhead) was located on the left side of the portal vein (white arrow) and on the ventral side of the inferior vena cava (white arrow). **d** The strings taping the proper hepatic artery and common hepatic artery (white arrow) were used for traction

**Fig. 5:**
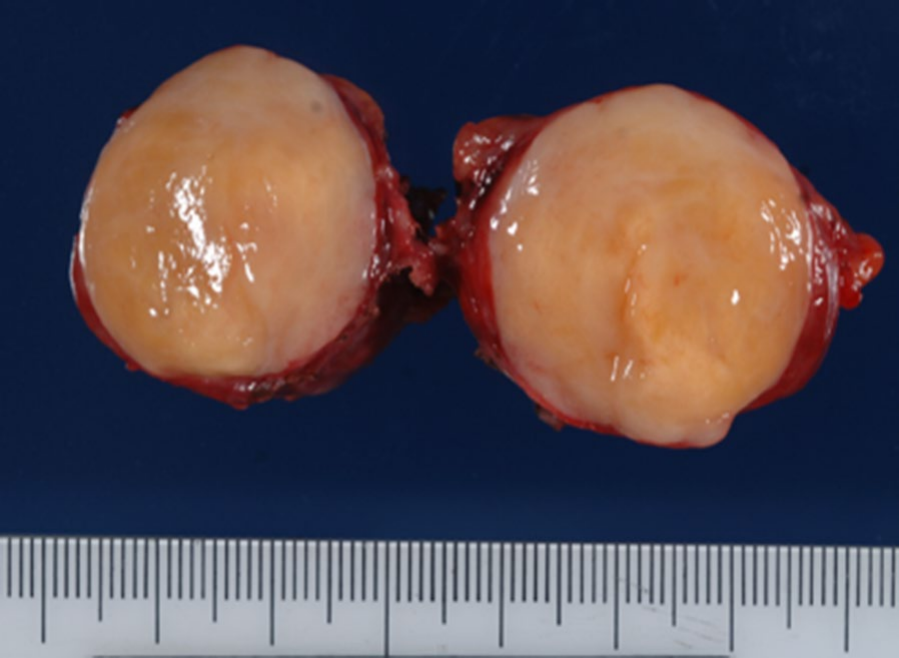
Macroscopic findings. The elastic, hard tumor measured 40 × 30 mm and had a smooth surface and fibrous capsule. The cross-section was milky white in color

**Fig. 6:**
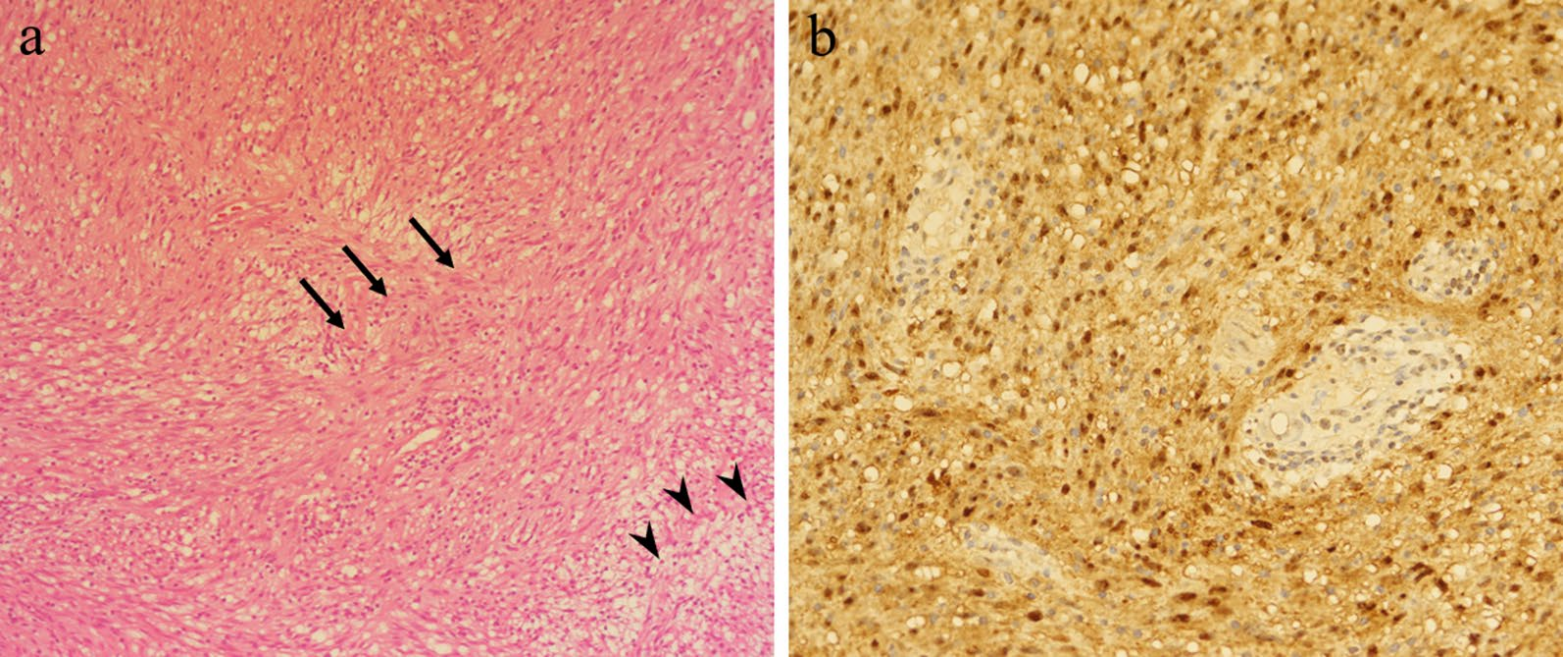
Histopathological findings. **a** The tumor was mainly composed of spindle cells and hypercellular (Antoni type A) (black arrow) and hypocellular (Antoni type B) (black arrowhead) areas (hematoxylin–eosin stain, original magnification × 400). **b** Immunohistochemical investigations revealed that the tumor was strongly positive for S-100 protein

A 54-year-old man was admitted to the Department of Surgery at our hospital following abnormal abdominal CT findings. He had no complaints, such as upper abdominal pain or abdominal distension. He presented with comorbidities of neurogenic bladder due to spinal cord injury and sleep apnea syndrome. His past history included previous surgeries for appendicitis and renal stones in the left kidney. The laboratory data showed normal findings. Abdominal contrast-enhanced CT revealed a tumor in the hepatoduodenal ligament, measuring 40 mm, with enhancement at its margin in the arterial phase; the enhancement was prolonged in the delayed phase (Fig. 1a, b). The boundary between the tumor and the caudate lobe of the liver was unclear. The abdominal magnetic resonance imaging (MRI) showed that the tumor was hypointense on T1-weighted images and both hypo- and hyperintense on T2-weighted fat-suppression images (Fig. 2a, b). Moreover, the contrast MRI showed that the margin of the tumor was enhanced in the arterial phase (Fig. 2c). The boundary between the tumor and the liver was clear; hence, the tumor was considered an extrahepatic lesion. Positron emission tomography (PET) revealed localized accumulation of fludeoxyglucose (FDG) (5.9 F) in the hepatoduodenal ligament tumor (Fig. 3). The biopsy was difficult to perform due to the location of the lesion. Although no preoperative diagnosis was made, surgical intervention was planned for an accurate diagnosis. The patient underwent 5-port laparoscopic surgery for tumor resection. Perioperatively, the tumor was found to be located on the left side of the portal vein and on the ventral side of the inferior vena cava (Fig. 4a–c). The tumor was surrounded by a fibrous capsule and did not infiltrate the other organs. In the operative technique used, the proper hepatic artery and common hepatic artery were taped at three sites (Fig. 4d). Each tape was used for traction in order to proceed with smooth and safe tumor resection. The total operative time was 273 min, and the total intraoperative blood loss was minimal. Macroscopic examination of the specimen showed an elastic, hard tumor measuring 40 × 30 mm with a smooth surface and fibrous capsule (Fig. 5). The cross-section of the tumor was milky white in color. Histopathologically, the peripheral nerve was near the tumor. The tumor was mainly composed of spindle cells and hypercellular (Antoni type A) and hypocellular (Antoni type B) areas (Fig. 6a). Immunohistochemical staining revealed that the tumor was strongly positive for S-100 protein (Fig. 6b), but had an extremely low Ki-67 positive rate. The patient was diagnosed with low-grade schwannoma. The postoperative course was uneventful, and the patient was discharged on postoperative day 5.

## Discussion

Most schwannomas are benign and account for approximately 5% of the benign soft tissue neoplasms [4, 5]. Although schwannomas often originate as solitary neoplasms, around 10% of them originate as multiple neoplasms [6]. The incidence rate of schwannomas is not related to sex or race. They are most commonly found in middle-aged patients (20–50 years old) [1, 4]. Some case reports have reported the occurrence of schwannomas in the abdominal viscera, such as the retroperitoneum [2, 7], stomach [3], gallbladder [8], pancreas [9, 10], bowel mesentery [11], colon [12, 13], and liver [14]. However, schwannomas in the hepatoduodenal ligament are extremely rare, and there are only few case reports on them. Since 1993, several reports have demonstrated schwannomas in the hepatoduodenal ligament, as shown in Table 1 [15–22]. The characteristics of nine cases with schwannoma in the hepatoduodenal ligament, including our case, were as follows: mean age, 49 years (range, 29–70 years) and female-to-male prevalence ratio, 1:2. Most cases were asymptomatic, and only one case reported the presence of two tumors.

**Table1:** Review of diagnosed cases of schwannoma in the hepatoduodenal ligament

Case	Author	Published years	Age	Sex	Symptoms	Number	Size (max), mm	Preoperative diagnosis	Treatment	Benign/malignancy	Follow-up (month)
1	Nagafuchi [15]	1993	62	F	Asymptomatic	Solitary	90	Hilar mass	Laparotomy	Benign	26
2	Pinto [16]	2011	29	M	Asymptomatic	Solitary	45	Spindle cell neoplasis or stromal tumor	Laparotomy	Benign	NA
3	Tao [17]	2016	50	M	Abdominal pain	Solitary	45	Stromal tumor	Laparoscopic surgery	Benign	7
4	Xu [18]	2016	43	M	Asymptomatic	Solitary	85	Abdominal mass	Laparotomy	Benign	NA
5	Liu [19]	2016	43	M	Abdominal pain	Two	45	Hilar mass	Laparotomy	Benign	8
6	He [20]	2020	70	M	Abdominal distension	Solitary	50	Abdominal mass	Laparotomy	Benign	17
7	Tomioka [21]	2020	38	F	Asymptomatic	Solitary	30	Spindle cell neoplasis	Laparoscopic surgery	Benign	NA
8	Wang [22]	2020	58	F	Right epigastric pain	Solitary	48	Abdominal mass	Laparotomy	Benign	37
9	Our case	2021	54	M	Asymptomatic	Solitary	40	Abdominal mass	Laparoscopic surgery	Benign	7

*M* male, *F* female, *NA* not available

Generally, contrast-enhanced CT findings of schwannomas show a hypodense mass with peripheral enhancement [23]. The MRI findings usually present as hypointensity on T1-weighted images and mixed hypointensity and hyperintensity on T2-weighted images [24, 25]. Schwannomas demonstrate accumulation of FDG on PET, and increased FDG uptake in the schwannomas is associated with malignancy [26, 27]. However, accurate preoperative diagnosis is difficult due to its nonspecific imaging characteristics and clinical symptoms. A definitive diagnosis of schwannomas can be made by histopathological and immunohistochemical examinations. Notably, only one report preoperatively diagnosed the schwannoma in the hepatoduodenal ligament by endoscopic ultrasound-fine needle aspiration [21]. The main components of the schwannomas include spindle-shaped cells and mixed hypercellular and hypocellular areas [14]. Such variation in cell density contributes to the heterogeneous and hyperintense images on T2-weighted MRI. Schwannomas test positive for S-100 protein and negative for desmin, smooth muscle actin, CD34, and CD117 [28, 29].

The main treatment of schwannomas is surgical resection. There was one case where a benign schwannoma turned malignant [28]. Notably, benign schwannomas rarely recur [30]. Complete excision without lymph node dissection is important for curative treatment [18, 31]. In 2016, Tao et al. [17] reported the first case treated by laparoscopic surgery. The previous case reports (Table 1) show that only three cases underwent laparoscopic resection, and their tumor sizes were less than 50 mm. In our case, a preoperative diagnosis was not made. We planned the laparoscopic approach to observe whether the origin of the tumor was intrahepatic or extrahepatic. During the surgery, laparoscopic surgery could be continued, because the tumor was surrounded by a fibrous capsule and no other organs were infiltrated. If there is infiltration in the surrounding organs, it is important to convert the laparoscopic surgery to laparotomy surgery for curative treatment. The traction of blood vessels surrounding the tumor proved extremely useful in expanding the surgical field. With the laparoscopic operative method, the surgery proceeded smoothly and safely.

## Conclusions

We encountered a rare case of schwannoma in the hepatoduodenal ligament that was laparoscopically resected. Depending on the clinical conditions, such as tumor size and no findings of infiltration in the surrounding organs, laparoscopic surgery for schwannomas in the hepatoduodenal ligament can be extremely useful.

## Data Availability

No applicable.

## Abbreviations

CT: Computed tomography
MRI: Magnetic resonance imaging
PET: Positron emission tomography
FDG: Fludeoxyglucose
